# Happiness Management: A Culture to Explore From Brand Orientation as a Sign of Responsible and Sustainable Production

**DOI:** 10.3389/fpsyg.2021.727845

**Published:** 2021-08-06

**Authors:** Rafael Ravina-Ripoll, Estela Nunez-Barriopedro, David Almorza-Gomar, Luis-Bayardo Tobar-Pesantez

**Affiliations:** ^1^Department of Business Management, Faculty of Economic and Business Sciences, INDESS, University of Cádiz, Cádiz, Spain; ^2^Economics and Business Management Department, Faculty of Economics, Business and Tourism, University of Alcalá, Alcalá de Henares, Spain; ^3^Salesian Polytechnic Department of Statistics and Operations Research, Faculty of Labour Sciences, University of Cádiz, Cádiz, Spain; ^4^Faculty of Economics, Salesian Polytechnic University, Cuenca, Ecuador

**Keywords:** happiness management, brand orientation, responsible, sustainable production, SEM, values, norms, behaviours

## Abstract

The overarching call to action represented by the sustainable development goals (SDGs) calls for new sustainable production and management models. Likewise, in periods of crisis, such as the current COVID-19 pandemic, companies are forced to develop competitive and sustainable development strategies to increase their brand value and achieve a good market position. Therefore, this work’s main objective is to design a structural equation modelling (SEM) to analyse the main critical dimensions of brand orientation to influence happiness in responsible and sustainable entities. For this purpose, a descriptive cross-sectional study was carried out based on primary data from a survey of a representative sample of 216 managers of small- and medium-sized enterprises in Andalusia (Spain) in the construction, industry and services sectors. The model results reveal significant dimensions of brand orientation and positively direct influence on happiness management. One of the significant managerial implications of this work is that the model allows for more responsible and sustainable management of entities by considering brand orientation about happiness.

## Introduction

Recently by mid-2020, COVID-19 has become a significant threat to the global economy and people’s quality of life and, consequently, their happiness. This environment is not at all conducive to the productive growth of companies, quite the contrary. As a result, many companies have been forced to take two strategic actions that would have been unthinkable before the Great Recession of 2008. The first is to drastically reduce their business activity due to the economic crisis brought on by this terrible pandemic. Moreover, the second is to undertake massive redundancy procedures that allow them to escape from their negative exploitation results at the expense of those who lose their jobs.

With these management actions and many others, top management wants to cushion their high economic losses in this perfect storm. In this new context, organisations’ brands play a vital role in securing their customers’ loyalty in the medium and long term.

This work is aligned with SDG 12, named ‘responsible production and consumption’. Whose aim is to study productive and economic models focused on sustainable development, brand orientation and corporate happiness. Precisely, the wellbeing of workers, consumers and society must be a premise in marketing strategies and the management of brand orientation. On the other hand, this work has implications from the consumer’s point of view. Consumers are changing their shopping behaviours with a growing trend towards sustainable brands, which is more efficient in happiness management and more social friendly.

Numerous contributions to the literature encourage SDG 12, ‘responsible production and consumption’, through the efficient use of resources (De Miguel Ramos and Laurenti, 2020; Zengin et al., 2021). However, one of the original contributions of this work to the literature and management is the consideration of ‘responsible production and consumption’: the efficient management of material resources and the design of a management model, in which behaviours, values and social norms contribute to the wellbeing and serve as a sign of brand identity. In this way, they create a corporate philosophy and culture that cares for the wellbeing of all the agents involved in the exchange relationship and contributes to the responsible development of a better world.

Branding is an essential intangible resource that companies possess to strengthen their corporate identity and differentiate themselves from their direct and indirect competitors (Wong and Merrilees, 2005). It is therefore not surprising that this issue has been examined since the end of the last century from the perspectives of economics, sociology, statistics and psychology, under the cross-cutting prism of perception, knowledge, loyalty, differentiation, marketing, social responsibility, communication and orientation (Keller, 2003; Santos-Vijande et al., 2013; Hirvonen and Laukkanen, 2014). Therefore, it makes sense for companies’ strategic direction to gravitate around brand orientation rather than other psycho-organisational factors (Urde, 1999). Thus, brand orientation is one of the main intangible assets that corporations have to invigorate their economic growth and organisational competitiveness (Yori Conill et al., 2011). In this sense, it should be noted that there are currently no governance models aligned with their brand orientation. Given this reality, the companies must understand that the brand orientation variable takes on a particular relevance to boost their customers’ subjective wellbeing and human capital significantly. In this way, companies will be better prepared to emerge from the red numbers brought about by the global COVID-19 pandemic (Ebersberger and Kuckertz, 2021).

The organisations’ generic strategies should implement happiness management’s attractive culture. A philosophy aligned with brand orientation would boost the brand position under the guiding principles of quality, social marketing, customer satisfaction, creative talent, work passion and transformational leadership (Sánchez-Vázquez and Sánchez-Ordóñez, 2019). From this approach, this research work aims to empirically examine the effects that the brand orientation parameter can have in happiness management on small- and medium-sized companies. Understanding happiness as a differential strategic factor allows companies to motivate the four letters I: intrapreneurship, technological innovation, organisational inclusion and emotional intelligence (Casaqui and Riegel, 2016).

In line with all that has been read here, it is worth noting that this article wants to contribute to the new wave of research emerging around the attractive topic of corporate happiness. Within this area of knowledge is the innovative organisational culture of happiness management. A business philosophy on there is little literature at present (Ravina-Ripoll et al., 2021b). Hence the scientific need to make multidisciplinary research on this novel concept from the triangle of marketing, sustainability and production (Brans, 2004). This fact means that many academic aspects of happiness management remain to be explored, including those factors directly linked to brand orientation in the COVID-19 era. A topic on which there is little information is because most of the studies written in recent years on brand orientation have been based on inferentially analysing the effects of this variable on business performance (Piha et al., 2021).

Under this approach, it is interesting to ask whether business management based on happiness management can substantially improve the corporate image of companies in today’s market. Given the above, and to further explore this attractive business culture, the essential purpose of this article will be to examine empirically how the brand orientation dimension influences happiness management, which will allow us to find out whether a strategic direction oriented towards collective happiness is a good attribute of competitiveness, sustainability and business success.

Having defined this research’s objective, this article is structured as follows from this introduction. The following section reviews the literature and sets out the working hypotheses, followed by a description of the methodology. The fourth section presents the main results and, finally, the discussions and conclusions.

## Conceptual Background and Hypotheses

Since the late twentieth century, many economists and marketers have become attracted to the topic of variable brand orientation (Baumgarth, 2010; Anees-ur-Rehman et al., 2016; Sepulcri et al., 2020). This concept is defined as an operational and functional strategy to be implemented by managing organisations to protect brand identity by focusing on their customers (Núñez-Barriopedro et al., 2021).

Given this reality, the brand orientation construct should not go unnoticed by researchers as an intangible factor that significantly enriches the innovation and economic vitality of organisations (Gromark and Melin, 2011). Thus, some authors have designed scales on this dynamic construct from both the internal and external perspectives. These include the questionnaires of Ewing and Napoli (2005), Wong and Merrilees (2008), Baumgarth (2010) and Piha and Avlonitis (2018). The latter questionnaire, which is the one to be used for this academic study, is characterised by examining the variable orientation based on the analysis of the following four factors: value (top management attitudes), norm (motivation), artefact (symbolic communication) and behaviours (corporate performance). In this sense, it should be noted that the choice of this particular questionnaire is because these four dimensions are pretty well associated with the theory of happiness management (Ravina-Ripoll et al., 2021a).

There is currently no extensive scientific literature on how brand orientation empirically affects sustainability and business performance (Gupta et al., 2020). In the face of this reality, solid academic evidence suggests that a solid corporate brand facilitates its identification and sustainable position in the global marketplace. It attracts the potential future customers (Cuesta-Valiño et al., 2020a; Foroudi et al., 2020).

Scientific publications currently addressing corporate happiness’s influence, subjective employee wellbeing or happiness management on the multidimensional brand orientation parameter are relatively scarce (Sepulcri et al., 2020). An exception to this is found in Tumasjan et al. (2020), where it is empirically shown that a positive climate within organisations positively influences the brand orientation of employees. In this context, the present study contributes to the literature on the importance of inferentially exploring the brand orientation and happiness management link as a critical success factor for operational and functional development from a marketing and organisational.

### Happiness Management

Scientific works on happiness management are relatively scarce in the literature of happiness economics. One reason for this phenomenon is that this line of research is still in its emerging stage. The growing interest in this line of research highlights that happiness is one of the main assets that managers of organisations possess to cultivate their employees’ subjective wellbeing (Basinska and Rozkwitalska, 2020; Núñez-Barriopedro et al., 2020). In this way, internal customers considerably increase their creativity, productive effectiveness, motivation, etc. All these factors and many others are very relevant to foster a positive atmosphere within organisations and increase their human capital’s job satisfaction (Suzete et al., 2019).

These are not trivial issues in times of economic crisis, such as the one currently caused by COVID-19. As is well known, in periods of severe economic recession, companies are forced to freeze or lower their workers’ wages (Yue and Cowling, 2021). In this context, there is extensive literature that suggests, on the one hand, that monetary income has a significant impact on the social wellbeing of human beings (Chumg and Huang, 2021). Moreover, on the other hand, that income has a significant impact on the wellbeing of human beings (Deaton, 2008; Lakshmanasamy, 2021; Lee and Ohtake, 2021). On the other hand, involuntary exits of people from the labour market hurt their happiness (Nikolova et al., 2020). In line with this research, economists and psychologists have focused on empirically examining the leadership-happiness association in the era of Industry 5.0. Many of these studies show that transformational leadership directly influences corporate happiness in organisations, especially in productive sectors linked to new technologies and health (Sudha et al., 2016). Under the umbrella of this literature body, other authors have quantitatively examined the psychological and managerial factors that have an individual or choral effect on employees’ happiness at work (Singh and Aggarwal, 2018). Examples include the following vectors: engagement, passion for work, es-threes, loyalty, etc. (Joo and Lee, 2017; Wang, 2017; Tandler et al., 2020; Núñez-Barriopedro et al., 2021).

Human resource theory shows that strategic management models that focus on people instead provide a holistic incentive for their internal customers’ job satisfaction and, therefore, their professional performance (Sattar et al., 2015). To improve workers’ subjective wellbeing, the companies should have a governance style that enhances organisational happiness through positive experiences, emotional pay, interpersonal relationships, etc. (Kaiser et al., 2008; Haar et al., 2019; Datu and Restubog, 2020).

Highlights that, despite the existence of a growing volume of academic monographs on happiness at work, in today’s digital society, there is not yet a large body of research on the topic (Adnan-Bataineh, 2019). There is currently little scientific evidence on how a corporate culture that revolves around the subjective wellbeing of its employees is a source of economic, financial and social profitability (Baker et al., 2006). One of the novelties of this work is designing a model in which the brand orientation achieves the happiness management construct. ‘A multicultural management model aimed at encouraging the following resources in job performance: creativity, commitment, technological innovation, internal entrepreneurship and social responsibility. In this way, organisations can cultivate the virtuous circle of corporate happiness’ (Ravina-Ripoll et al., 2019).

### Hypothesis Statement

**Figure 1:**
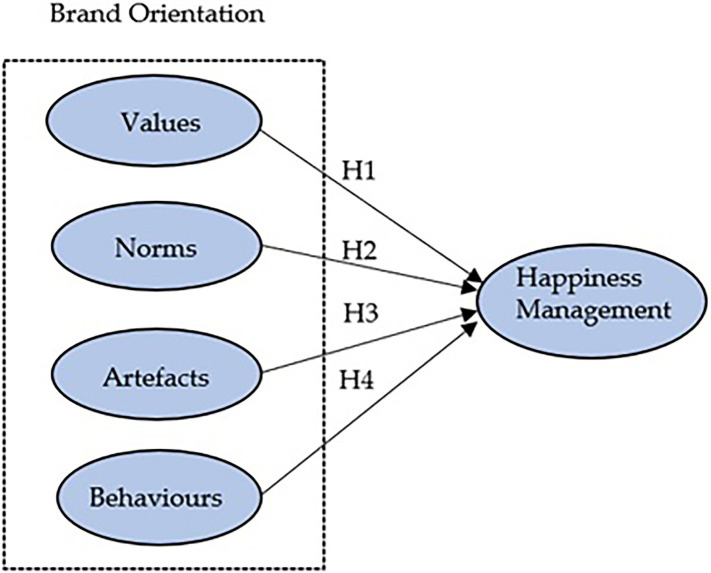
Conceptual model and hypothesis.

This sub-section explains the conceptual model (see Figure 1) with each of the research hypotheses. The SEM model allows explaining each of the dimensions of the brand orientation construct and its relationship with happiness management in the context of small and medium enterprises. In line with the objectives of GDS 12, we cannot forget to mention that many recent studies highlight the significant number of companies implementing management models that ensure sustainable consumption and production at a global level (Goyal et al., 2021; Yu et al., 2021). These executive actions are in line with the principles of the circular economy, which now constitute a substantial range of opportunities for companies to cultivate sustainable and lasting competitive advantages over time (Camilleri, 2020; Camana et al., 2021).

**Figure 2:**
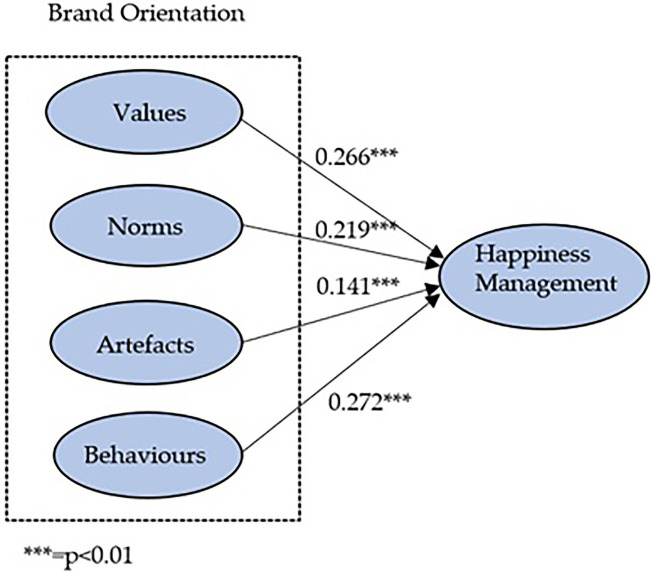
Graphical representation of the structural equation model.

In this sense, it is not surprising that today’s managers of large multinationals are beginning to consider brands as an intangible asset on which companies and their environmental and organisational marketing culture should be based (Napoli, 2006). This issue cannot be dealt with in-depth in this academic text because it would go beyond the limits of this article. However, the association between brand orientation and happiness management, as shown in the following illustration, together with the hypotheses put forward in this research, can be dealt with in-depth.

In this respect, Aaker (2012) and other authors point out that brands should maximise customer satisfaction through the prism of loyalty, excellence, and the quality of their products or services (Cuesta-Valiño et al., 2019). In this way, companies can exponentially increase their competitive position in the global marketplace and thus their economic performance in both the medium and long term. The brand orientation parameter is directly linked to stakeholders under the guiding principles of usefulness, added value, functionality, differentiation and symbolic capabilities (Hankinson, 2002).

Therefore, the following study hypothesis is put forward.

*H1*: The level of values (brand orientation) positively influences the level of happiness management.

Therefore, organisations should encourage this type of management policy to impact consumers’ perception of their products or services positively. Marketing managers should propose to their superiors to implement attractive functional and operational strategies that connect directly with their corporate brands’ mission, vision and values (Odoom and Mensah, 2019; Foroudi, 2020). It is particularly relevant in today’s globalised market. Large multinationals generate a wellspring of loyalty to their brand through identity, happiness, social commitment, image, neuromarketing, trust, etc. All of this undoubtedly contributes to customers becoming emotionally loyal to the brand rather than reasoning (Gupta et al., 2008). These aspects mean that the brand value is associated with the company’s market value (Sheth, 2021). It means that the social and functional attributes of brands become an essential lever for improving their competitiveness. It allows them to differentiate themselves from their competitors in quality, loyalty, reputation, innovation, etc. It means that the social and functional attributes of brands become an essential lever for improving their competitiveness. It allows them to differentiate themselves from their competitors in quality, loyalty, reputation, innovation, etc. (Chang et al., 2018).

Therefore, the following study hypothesis is put forward:

*H2*: The level of standards (brand orientation) positively influences the level of happiness management.

Other researchers suggest that brand orientation can significantly boost potential customers’ purchase intent. This fact takes on particular relevance in times of severe economic or health crises, such as the one we are currently experiencing (Dash et al., 2021). To achieve this, brand identities (mission, vision and values) must be integrally involved with companies’ organisational culture and strategic plans (Urde et al., 2013). It is not possible, in managerial terms, without the existence of inter-organisational ecosystems that stimulate the following factors in all members of the organisations: motivation, learning, eco-innovation, disruptive thinking, autonomy, creative talent and happiness (Dechawatanapaisal, 2018; Khan and Bashir, 2020; Sanagustín-Fons et al., 2020). In general, this corpus of intangibles and many others are not very highly valued by the managers of small- and medium-sized companies in times of economic crisis. These intangible assets are essential to driving their productivity. For their brands to achieve brand equity (Wirtz et al., 2013), this implies that brand orientation must be seamlessly integrated with the organisation’s strategic direction (Tajeddini and Ratten, 2020). In this way, companies can exponentially improve, on the one hand, their sustainable and competitive positioning in the market (Vallester and De Chernatony, 2006). On the other hand, companies can significantly increase their business profit. It must be accompanied by an applicable policy that pivots around the construct of brand orientation as a sign of quality, loyalty and social marketing (Avlonitis and Salavou, 2007; Shih, 2018).

Thus, the following study hypothesis is put forward:

*H3*: The level of artefacts (brand orientation) positively influences the level of happiness management.

Many articles written on the economics of happiness throughout the twenty-first century are notable for using the word happiness in their studies as a synonym for subjective wellbeing, job satisfaction or quality of life (Mackerron, 2012; Oishi, 2012; Seligman, 2016). A cognitive resource facilitates researchers in approaching the theoretical and quantitative analysis of the construct of happiness management (Jiménez-Marín et al., 2020). Some of these studies show that companies’ best way to design a competitive, innovative, proactive and sustainable organisational architecture is happiness management. It is a corporate philosophy and operation that enhances the brand image and its relationship with consumers (Ravina-Ripoll et al., 2021b). To this end, it makes sense for companies to undertake human resources policies aimed at conveying constructive values, positive emotions or happiness. They are bearing in mind that brand orientation is crucial for the achievement of this goal. Thus, companies must adopt policies to transmit constructive values, positive emotions or happiness 5.0 (Schöler, 2019) to implement happiness management in the industry successfully.

Thus, the following study hypothesis is put forward:

*H4*: The level of behaviour (brand orientation) positively influences the level of happiness management.

## Methodology

This paper’s main objective is to analyse the structural relationship between brand orientation and happiness management in the business environment of the COVID-19 era. This research is descriptive, randomised and exploratory because it examines the predictive causal relationship between the two dimensions. The surveys were sent online to the managers of Andalusian small- and medium-sized enterprises registered in the Andalusian Institute of Statistics, using the digital tool Google Docs. The data collection methodology has been a random sample without replacement. Once all the responses were obtained from the submitted surveys, the database was then processed and statistically analysed with the statistical software SPSS 27 (see Table 1).

**Table 1:** Technical specifications of the sample.

Data collection methodology	Random sample without replacement
Universe	Small- and medium-sized enterprises in Andalusia (Spain)
Unit of analysis	Managers of small- and medium-sized enterprises in Andalusia (Spain)
Population under study	9.308 managers of small- and medium-sized enterprises in Andalusia (Spain)
Sample size	216 executives of small- and medium-sized enterprises in Andalusia (Spain)
Sampling error	±4.9% (95% confidence level)
Period of analysis	September–December 2020

The sample comprises 216 people with the profile of managers of small- and medium-sized companies in Andalusia (Spain), whose main activity is the service sector (42.6%). In addition to this information, the interviewees’ average age is 42.86 years (standard deviation of 8.8 years). It should be noted that 85.65% of those surveyed were male, and 88.88% of the total had a university degree. This sample shows that less than a third of the participants (22.69%) have been managers in these organisations for more than five years and that 95.82% of the managers surveyed work in companies with fewer than 25 employees (see Table 2).

**Table 2:** Characteristics of the sample.

	Frequency absolute	Percentage
***Sector***		
Construction	55	25.46
Industrial	69	31.94
Services	92	4.6
***Number of employees***		
1–5	140	64.81
6–15	52	24.07
16–24	15	6.94
*>25*	9	4.18
***Gender***		
Female	31	14.35
Male	185	85.65
***Level of study***		
PhD	1	0.46
Master’s degree	15	6.94
University studies	192	88.88
Secondary education	8	3.72
***Seniority in the company***		
<1 year	5	2.31
1–5 year	162	75
>5 year	49	22.69
***Age***		
Minimum	28.75	
Maximum	60.09	
Mean	42.86	
Standard deviation	8.8	

The questionnaire was implemented online in the last quarter of 2020 in Spain, i.e. in the middle of the pandemic. In this regard, it should be noted that these companies’ managers were informed before starting to fill in the questionnaire. On the one hand, that participation was voluntary. On the other hand, they declared that they were fully informed about the scientific purpose of this research. The questionnaire consists of twenty-five variables (see Table 3). The Baumgarth brand orientation scale was used for this purpose (Baumgarth, 2010; Baumgarth et al., 2013), which measures this construct based on the sub-items: values, norms, artefacts and behaviours (Wong and Merrilees, 2007; Guzmán-Maldonado et al., 2019). The scale comprises nineteen questions on a Likert scale with a range of values from 1 ‘strongly disagree’ to 5 ‘strongly agree’. The variable of happiness is also considered (Foncubierta-Rodríguez et al., 2020). Thus, the two most commonly used items to measure this parameter were selected (Núñez-Barriopedro et al., 2020). In addition to these two questions, four other questions were added relating to turnover, profitability, operating result and profit on a Likert scale of 0 to 5 points (AECA, 2005). The inclusion of these items is motivated by the fact that the answers given to these questions allow us to know the companies’ competitiveness. It must not be forgotten that happy organisations are synonymous with economic profitability and productive efficiency (Baker et al., 2006).

**Table 3:** Measurement models of constructs, items and factor loadings.

Construct	Code	Factor loadings	Items	Sources of adoption
Values	VM1	0.968^***^	Decisions around the branding of our products or services are discussed at the senior management level.	Baumgarth, 2010; Gromark and Melin, 2011; Baumgarth et al., 2013
	VM2	0.9692^***^	Our brands are differentiated from those of our competitors.	
	VM3	0.954^***^	We ensure that our brand positioning is maintained over the long term.	
	VM4	0.937^***^	We seek to ensure that our brand management is consistent over long periods.	
	VM5	0.904^***^	We invest in our product or service brands even in times of scarce financial resources.	
Norms	NM1	0.879^***^	We regularly check that the corporate design guidelines of our brands are adhered to.	Wong and Merrilees, 2008; Baumgarth, 2010; Baumgarth et al., 2013
	NM2	0.851^***^	In all information issued about our brands, explicit attention is paid to the integration of communication methods.	
	NM3	0.866^***^	We have detailed written specifications of the brand positioning of our products or services.	
	NM4	0.833^***^	We have managers who have clear responsibility for the branding of our products or services.	
	NM5	0.772^***^	Brand managers have the competence and authority to succeed with the positioning of our brands.	
	NM6	0.741^***^	We regularly check that our brands are differentiated from those of our competitors.	
Artefacts	AM1	0.964^***^	Our employees visibly display our product or service brands (e.g. uniforms and shirts).	Baumgarth, 2010; Baumgarth et al., 2013
	AM2	0.972^***^	Our stands at trade fairs in which we participate always reflect the brands of our products or services.	
	AM3	0.975^***^	We hold regular meetings on the current status of our product or service brands.	
	AM4	0.964^***^	We have ‘Stories’ in the company that reflect the positioning of our brands.	
Behaviours	CM1	0.951^***^	We invest in advertising the company’s image.	Baumgarth, 2010; Baumgarth et al., 2013
	CM2	0.929^***^	We teach our employees about the importance of our brands.	
	CM3	0.889^***^	We educate new employees on the positioning of our brands.	
	CM4	0.810^***^	We regularly conduct market research on our brands.	
Happiness management	HM1	0.601^***^	What would you consider your company’s return on investment (ROI) to have been over the last two years?	Foncubierta-Rodríguez et al., 2020; Núñez-Barriopedro et al., 2020
	HM2	0.622^***^	What would you consider your company’s profit level to have been over the last two years?	
	HM3	0.845^***^	What has been your company’s sales volume over the last two years?	
	HM4	0.921^***^	To what extent do you consider your customers to be happy with your company’s services or products?	
	HM5	0.929^***^	To what extent do you consider yourself a happy or unhappy person in your company?	

*** *Indicates 99% significant*.

The procedure followed for the analysis of the results is that of structural equation modelling. This statistical technique allows researchers to establish *a priori* the specific relationships between variables (Batista-Foguet et al., 2004). SPSS 27 statistical software was used for this purpose.

## Results

Cronbach’s alpha coefficient was calculated to explore the multidimensional robustness of the questionnaire used to analyse the dimensions of brand orientation (values, norms, artefacts and behaviours) and happiness management (see Table 4). This test statistic is higher than 0.7 points (*p* < 0.01). For Hair et al. (1995), a value higher than this figure indicates the reliability of the scales used (see Table 4).

**Table 4:** Consistency and reliability analysis.

	Cronbach’s alpha	Composite reliability (CR)	The average variance extracted (AVE)
Values	0.972	0.968	0.901
Norms	0.923	0.918	0.681
Artefacts	0.979	0.933	0.923
Behaviours	0.94	0.941	0.805
Happiness management	0.915	0.911	0.651

However, the extensive literature on descriptive statistics shows that Cronbach’s alpha coefficient lacks robust statistics. This fact means that it is impossible to state the absence of other latent variables in our model and multicollinearity (Celina-Oviedo and Campo-Arias, 2005; Abu-Bader, 2021). One of the reasons for this is that one of this coefficient’s assumptions is the continuous nature of the variables (Elosua and Zumbo, 2008) when an ordinal response scale is usually used.

Two inferential tests are carried out to achieve the purpose. The first, the Composite Reliability (CR) proposed by Bagozzi and Yi (1988). This indicator gives a value of more than 0.7 points (*p* < 0.01), which is higher than the register indicated by the researchers above for asserting the reliability of our scales. The second, the Index of Variance Extracted (IVE), whose value is higher than 0.5 points (*p* < 0.01). So, a digit higher than this figure allows us to affirm the existence of a solid empirical consistency of the constructs that are the object of this study (Fornell and Larcker, 1981).

The theoretical model’s discriminant validity has been ensured using the confidence interval test proposed by Anderson and Gerbing (1988). The authors above indicate that there is validity at a 95% confidence level when the correlations between constructs are significantly lower than unity. All constructs satisfy this criterion. Likewise, the correlations of the variables in our model are relatively high. The correlation between norms and values is the highest (0.933), which seems consistent due to their importance in corporate culture, followed by that between artefacts and values (0.845) and behaviour with values (0.747). Norms are also strongly associated with artefacts (0.738) and the dimensions norms and behaviours (0.726), as shown in Table 5.

**Table 5:** Correlations between constructs.

	Values	Norms	Artefacts	Behaviour	Happiness management
Values	1				
Norms	0.901^**^	1			
Artefacts	0.933^**^	0.686^**^	1		
Behaviour	0.845^**^	0.738^**^	0.944^**^	1	
Happiness management	0.747^**^	0.726^**^	0.486^**^	0.807^**^	1

** *p < 0.01*.

Next, the correlations of the factors that make up the brand orientation dimension with the happiness management construct are analysed. The results show that the happiness management construct is correlated with the vectors that make up the brand orientation variable in a sensible way, with the lowest relationship between values and happiness management.

Based on the numerical data described in Table 4, a confirmatory factual analysis was carried out using the maximum likelihood approach (Lio and Liu, 2020). The results show the existence of a fairly acceptable goodness of fit of our structural conceptual model (S-BX2 = 1.554171; gl = 265; *p* = 0.000; NFI = 0.854; NNFI = 0.869; CFI = 0.876; RMSEA = 0.079). Thus, there is a good fit between the respondents’ answers and our conceptual model described in Figure 1.

In the light of this finding, a structural equation model is carried out to verify the influence of brand orientation on the happiness management construct. The results obtained on the significance of this paper’s hypotheses reveal that the factors that make up the dimensions of brand orientation have positive and significant effects on the happiness management vector. It means that all our working hypotheses (H1, H2, H3 and H4) are supported.

In this sense, we note that the behaviour factor is the most influential factor on the happiness management construct (*β* = 0.272, value of *p* 0.01). The minor empirical significance is the artefact variable (*β* = 0.141, value of *p* 0.01). Therefore, it can be affirmed that brand orientation has a positive effect on the happiness management construct. It can be seen empirically in their structural equation system (Happiness Management = 0.266 Values + 0.219 ×Norms + 0.144×Artifacts + 0.272 Behaviours), as well as descriptively in the following illustration (see Figure 2).

Given this statistical information, it is observed that a strategic direction that revolves around brand orientation generates positive synergies on organisations’ corporate happiness in COVID-19. In general terms, this research coincides with our four study hypotheses and the literature review on the happiness management construct (Ravina-Ripoll et al., 2019; Silva Munar et al., 2020).

## Discussion

The sustainable development goals (SDGs) require new models of responsible and sustainable production and management. Therefore, one of the main objectives of this work is to define a model of responsible and sustainable organisations that contributes to the literature and has implications for all actors in the value chain. On the one hand, through a brand-oriented model, organisations can be more responsible and sustainable, and on the other hand, they can be sustainable, and consequently pursue corporate happiness (Harju et al., 2021). On the other hand, from a demand-side point of view, it allows consumers to manage the time spent on purchasing choices by choosing responsible and sustainable brands and even feel more satisfied by contributing to sustainability (Currás-Pérez et al., 2009).

### Theoretical Implications

The different authors prioritise different elements, such as creating and appropriating value and the increase in profitability. However, we can affirm that a sustainable organisation model must be an integral structure, covering all the elements that have been mentioned holistically (Zott et al., 2011).

The sustainability of an organisation depends mainly on the organisation’s brand orientation, which is related to achieving an adequate structure and capabilities to execute its strategic decisions that influence corporate happiness. This organisational sustainability process involves continuous adaptation and change about its strategy and business model (Osterwalder et al., 2005). Furthermore, the models require creating added value for customers to gain a competitive advantage (Chauhan et al., 2021).

Thus, the term ‘brand orientation’ is mainly based on four pillars: value, norms, artefact and behaviours which are connected and complement each other, giving meaning and raison d’être to the concept in question, representing a cycle in which a responsible and sustainable production model (González-Mansilla et al., 2019). Value, according to the findings, it is essential (0.9692) that brands are differentiated from competitors; (0.968) that product branding decisions are discussed at the senior management level; (0.954) that brand positioning is maintained over the long term; (0.937) that brand management is consistent over long periods; and (0.904) that we invest in our product or service brands even in times of scarce financial resources. Therefore, senior management should build long-term brand equity to allow for differentiated positioning (Baumgarth, 2010; Baumgarth et al., 2013). Norms, according to the findings, in this dimension of brand orientation play an essential role (0.879) regularly checking that the brands’ corporate design guidelines are adhered to; (0.866) written specifications of brand positioning; (0.851) all information issued about the brands, explicit attention is paid to the integration of communication methods; (0.833) managers have clear responsibility for the brand; (0.772) brand managers have the competence and authority to carry out the positioning of their brands successfully, and (0.741) they regularly check that brands are differentiated from competitors.

Therefore, brand information should be aligned with brand design, organisational communications and positioning. An organisation’s brand is differentiated from the competition through strategies related to corporate happiness (Baumgarth et al., 2013). Artefact, according to the findings, in this dimension of brand orientation, plays an important role (0.964) to hold regular meetings on the current status of the brands that stands at trade fairs should reflect the brands; (0.964) that during all contact with customers, employees should visibly display brand elements of products or services; and (0.964) to have ‘Stories’ in the company that reflect the brand positioning. Therefore, it involves showing corporate identifications offline and online, from brand elements in merchandising or social media stories. All this makes it interesting to design some corporate happiness seals similar to quality certifications that help position the brand (Baumgarth 2010). Behaviours, according to the findings, in this dimension of brand orientation, the following has a significant role to play (0.951) to invest in advertising the company’s image, to teach employees about the importance of the brand, (0.889) to educate new employees on the positioning of the brand and (0,81) to regularly conduct market research on own brands. Companies must invest in their brand image and make it known to their employees given the above. It cannot be done without regular market research to enable companies’ brands to adapt to new changes in society. It will guarantee its sustainability in the long term (Wong and Merrilees, 2007; Mai et al., 2021).

In terms of ‘happiness management’, the findings show that (0.929) consideration of feeling a happy person in your company; (0.921) consideration of your customers being happy with your company’s products; (0.845) consideration of your company’s sales volume in the last two years; (0.622) consideration of the level of profits; and (0.601) consideration of the company’s return on investment (ROI) play an essential role. Therefore, the wellbeing of the company’s employees and customers is just as important, if not more so, than profit and profitability in companies that differentiate themselves through responsible production and consumption (Jiménez-Marín et al., 2021).

### Managerial Implications

Given that the environment is constantly changing, it must be frequently revised to better adapt to the environment by creating added value for business management and sustainable development. It is convenient to create new ideas unique to obtain a competitive advantage and enhance the brand value. Through the capacity and ability to achieve a brand orientation that influences corporate happiness, companies achieve success in their business (Sjödin et al., 2020).

Thus, the main objective of the research is to understand how organisations, through brand orientation, promote new sustainable models that allow them to have competitive advantages over their competitors. Moreover, thus, they occupy a favourable position in the market (Adnan-Bataineh, 2019; Wang and Zhang, 2020) by differentiating themselves through responsible production and consumption and investing in the wellbeing of the company’s employees and customers.

Brand orientation is also a fundamental component in the construction of current sustainable models. It is based on the fact that it will only generate value if it has a differentiating element within the market and strengthen corporate identity to gain brand equity (Shahiduzzaman et al., 2018).

The findings show a model of brand orientation based on four key factors that can serve as a strategy for brand differentiation and brand equity. The first dimension, ‘Value’, allows top management to adopt marketing strategies to increase responsible and sustainable value (Cuesta-Valiño et al., 2020b). Also, the needs of stakeholders are being met (Hankinson, 2002). The second dimension is the ‘Norm’; marketing managers can design strategies that connect directly to their corporate brands’ mission, vision and values (Odoom and Mensah, 2019; Foroudi, 2020). The third dimension, ‘Artefact’, brand identities (mission, vision and values) must be significantly related to the organisational culture and strategic plans of companies (Urde et al., 2013). The fourth dimension, ‘Behaviour’, implies that internal customers significantly increase their creativity, productive efficiency and motivation in responsible and sustainable organisations that guarantee the happiness of their employees. All of this increases the job satisfaction of their human capital and ensures the wellbeing of consumers (Yue and Cowling, 2021).

In this paper, corporate happiness is understood as a differential strategic factor motivating intrapreneurship, technological innovation, organisational inclusion and emotional intelligence (Casaqui and Riegel, 2016). We are aware that the latter concept has recently aroused growing interest in scientific research in the field of economics and business (Ravina-Ripoll et al., 2019).

There is no single business model that is competitive because they have different capacity business model systems. There is a connection between the formulation of an idea and implementing a natural, sustainable strategy. In this sense, organisations should design sustainable business models with differentiated elements from the competition.

Moreover, companies producing and launching their products beyond borders are changing significantly due to the economy’s birth and rapid development (Serra-Cantallops et al., 2021). In this new context, organisations’ brands play a vital role in organisations’ sustainability, thus ensuring their customers’ loyalty in the medium and long term (Gatto and Re, 2021; Grijalvo Martín et al., 2021).

### Limitations of the Study and Future Lines of Research

One of the limitations of this study is that it is a cross-sectional study. In future work, a longitudinal study with panel data can be carried out. The scope of the present study is Spain. In future work, it could be extended to a more international scope, applying to other countries. Another limitation of the study is the sample size. Future research could extend the sample to incorporate new regions to extrapolate and minimise sampling error.

Furthermore, the structural equation model analysed in this work is a first-order formative model. The brand orientation construct has been studied with its dimensions and its influence on corporate happiness for the responsible and sustainable development of organisations. So that new constructs can be added to the model in future work.

The main focus of the existing literature on brand orientation is on the brand orientation concept (Simões and Dibb, 2001; Urde et al., 2013) on internal brand management on brand performance (Wong and Merrilees, 2008; Baxter et al., 2013), among others but one of the novelties of this study is the multidimensional approach to brand orientation and the direct relationship of each of these dimensions with happiness management. Furthermore, this paper aims to contribute to the literature with a first-order model to lay the foundations of brand orientation from a new perspective of happiness management. Therefore, based on this work, future research can be developed. Second-order structural equation models can be developed, and the indirect effects of the variables can be measured (Frey, 2020).

Future research should empirically explore this construct in other types of organisations, such as nonprofit organisations. It is bearing in mind the academic expectations that currently exist in social sciences about the innovative concept of happiness management and its link to brand orientation and marketing (Serra-Cantallops et al., 2021).

## Conclusion

This research shows how brand orientation, in which values, norms, artefacts and behaviours serve as a sign of brand identity and contribute to the well-being of the brand. Thus, the value in brand orientation allows differentiating the brand from competitors in the long run, which positively influences happiness management (Balmer, 2013; Baumgarth et al., 2013). Value in brand orientation positively influences consumer identification (Tuškej et al., 2013). In this dimension of brand orientation, standards play an essential role by regularly checking compliance with corporate design guidelines of brands. In this respect, the integration of communication methods for brand promotion and their relationship with happiness management is essential. In this dimension of brand orientation, standards play an essential role in regularly checking compliance with the brands’ corporate design guidelines. The artefact plays a vital role in brand orientation by holding regular brand status meetings. It should visibly display the brand elements of the products during all contact with customers, employees. You must have ‘Stories’ in the company that reflect the brand positioning. It positively influences happiness management. In this dimension of brand orientation, behaviours have an essential role in investing in advertising the company’s image, teaching employees the importance of the brand and conducting regular market research on their brands. It will ensure the long-term sustainability of happiness management (Wong and Merrilees, 2007; Mai et al., 2021).

According to these results, where a significant relationship has been found between the two dimensions of this study, the model proposed here may constitute the way to design future management models based on brand orientation and happiness management (Bettiga and Lamberti, 2020). On the one hand, it can be achieved by constructing responsible, happy, creative and sustainable production models (González-Mansilla et al., 2019) and moreover, on the other hand, with the existence of a significant volume of professional managers involved in the belief that the corporate happiness of organisations is a strong differentiating element of their brand concerning their competitors (Cuesta-Valiño et al., 2020b).

Finally, the findings of this work may be a clear sign that consumers are increasingly demanding more sustainable products and companies that are guided by ethical principles of environmental responsibility and job satisfaction. In our view, such information can enrich the idea that organisations in the Industry 4.0 era understand happiness management as a strategic, innovative and transformative factor within the SDGs 12 (Foncubierta-Rodríguez et al., 2021).

## Data Availability Statement

The raw data supporting the conclusions of this article will be made available by the authors, without undue reservation.

## Author Contributions

RR-R, EN-B, DA-G, and L-BT-P: conceptualization, methodology, software, validation, formal analysis, investigation, resources, data curation, writing—original draft preparation, writing—review and editing, and supervision. All authors have read and agreed to the published version of the manuscript.

## Conflict of Interest

The authors declare that the research was conducted in the absence of any commercial or financial relationships that could be construed as a potential conflict of interest.

## Publisher’s Note

All claims expressed in this article are solely those of the authors and do not necessarily represent those of their affiliated organizations, or those of the publisher, the editors and the reviewers. Any product that may be evaluated in this article, or claim that may be made by its manufacturer, is not guaranteed or endorsed by the publisher.
